# Red foxes (*Vulpes vulpes*) and raccoon dogs (*Nyctereutes procyonoides*) as potential spreaders of *Sarcocystis* species

**DOI:** 10.3389/fvets.2024.1392618

**Published:** 2024-06-05

**Authors:** Ondřej Máca, Naglis Gudiškis, Dalius Butkauskas, David González-Solís, Petras Prakas

**Affiliations:** ^1^Department of Pathology and Parasitology, State Veterinary Institute Prague, Prague, Czechia; ^2^Department of Zoology and Fisheries, Faculty of Agrobiology, Food and Natural Resources, Czech University of Life Sciences Prague, Prague, Czechia; ^3^Nature Research Centre, Vilnius, Lithuania; ^4^Department of Systematics and Aquatic Ecology, El Colegio de la Frontera Sur, Chetumal, Mexico

**Keywords:** red fox, raccoon dog, Czech Republic, farm animals, molecular characterization, Protozoa

## Abstract

**Background:**

*Sarcocystis* includes a global group of apicomplexan parasites with two-host life cycle frequently circulating in wildlife and domestic hosts, including humans. Two of the most important wild terrestrial carnivores acting as definitive hosts are the red fox and raccoon dog, due to their wide distribution in Europe and usage of wild and farmed animals as prey. This study was conducted to determine the prevalence of *Sarcocystis* in hunted red foxes and raccoon dogs from nine regions of the Czech Republic and to identify isolated sporocysts by molecular techniques.

**Methods:**

Approximately 5 g of the contents of large intestine from 200 animals (197 red foxes and three raccoon dogs) were examined by flotation centrifugation coprological method. Only samples of 50 red foxes and one raccoon dog positive to *Sarcocystis* spp. were used for the nested PCR (nPCR) method to amplify a fragment or partial sequence on the *cox1* gene. Ten species-specific primer pairs for detection of *Sarcocystis* spp. using farm animals as intermediate hosts were utilized.

**Results:**

In total, 38.1% of the red foxes and 66.7% of the raccoon dogs were positive to *Sarcocystis* by light microscopy. The molecular characterization resulted in the identification of five species in the red fox: *S*. *arieticanis*, *S*. *capracanis*, *S. cruzi*, *S*. *miescheriana*, and *S. tenella*, while the PCR was negative for the sole raccoon dog. The highest intraspecific variation was found for *S*. *miescheriana*, while *S. tenella* was the most prevalent. Co-infections occurred in the large intestine of the red fox. No zoonotic species were found in our samples.

**Conclusion:**

This is the first study where the potential role of the red fox and raccoon dogs as spreaders of *Sarcocystis* to farm animals in the Czech Republic is shown. The use of species-specific primers provides a fast and easy method for screening multiple samples for a particular *Sarcocystis* species.

## Introduction

Members of the genus *Sarcocystis* are apicomplexan parasites of reptiles, birds and mammals including humans (1). They have compulsory two-host prey–predator life cycle. Asexual sarcocysts are found mainly in muscle tissues of intermediate hosts (herbivores, omnivores, and carnivores), while sexual sporocysts develops in the lamina propria of the small intestine of definitive hosts (carnivores, scavengers) (1–3). Definitive hosts get infected through consumption of animal tissues containing mature sarcocyst, while intermediate hosts acquire *Sarcocystis* infection via food or water contaminated with sporocysts. Some of *Sarcocystis* spp. (e.g., *S. canis*, *S*. *calchasi*, *S*. *falcatula*, *S*. *neurona*) are highly pathogenic for domestic and wildlife animals (1, 4–6). Furthermore, the livestock industry suffers losses due to macroscopic sarcocysts, reduced quality of meat due to intensive *Sarcocystis* infections or due to the rarely encountered clinical symptom induced by acute infections (1, 7, 8).

Morphological distinguishment of *Sarcocystis* spp. according to the sexual stages of the parasites is virtually not possible in the final hosts (1, 2, 9, 10). Therefore, definitive hosts of *Sarcocystis* spp. have historically been identified through laboratory transmission experiments (11–14). However, co-infection with several *Sarcocystis* spp. is very common in wild and domestic ungulates, which complicates the implementation and reliability of transmission experiments (15, 16). Furthermore, the ethical considerations related to the use of wild predatory mammals or birds make it crucial to find other approaches in revealing the life cycles of these parasites. Therefore, DNA analysis methods are now increasingly used to identify *Sarcocystis* spp. in intestinal or fecal samples of definitive hosts (9, 10, 17–23).

Representatives of the family Canidae (e.g., red fox [*Vulpes vulpes*], Arctic fox [*Vulpes lagopus*], coyote [*Canis latrans*], gray wolf [*Canis lupus*], raccoon dogs [*Nyctereutes procyonoides*], jackal [*Canis aureus*], dingo [*Canis lupus dingo*] and dog [*Canis lupus familiaris*]) are involved in the prey–predator life cycle of numerous *Sarcocystis* spp. Most of these parasite species employ domesticated and wild ungulates as their intermediate hosts (1). The red fox is widely distributed throughout the Northern Hemisphere (24) and suggested as the definitive host of about 20 *Sarcocystis* spp. forming sarcocysts in muscles of domestic and wild ungulates, small mammals, and birds (1, 19, 25–27). The raccoon dog primarily originated from the Far East, although nowadays it is one of the most prevalent invasive mammal species in Europe (28). It has been shown that raccoon dogs serve as definitive hosts of several *Sarcocystis* spp. employing roe deer (*Capreolus capreolus*), reindeer (*Rangifer tarandus*), pigs and wild boar (*Sus scrofa*), and ducks (*Anas platyrhynchos*) as their intermediate hosts (18, 26, 29). However, there is a lack of molecular or epidemiological investigations addressing the role of red foxes and raccoon dogs in the transmission of *Sarcocystis* spp. to farm animals. Since the red fox and raccoon dog serve as definitive and reservoir hosts for a wide variety of *Sarcocystis* spp., the main goal of the present study was to determine the *Sarcocystis* spp. using these canid hosts as definitive hosts and farmed animals as intermediate hosts.

## Methods

**Figure 1:**
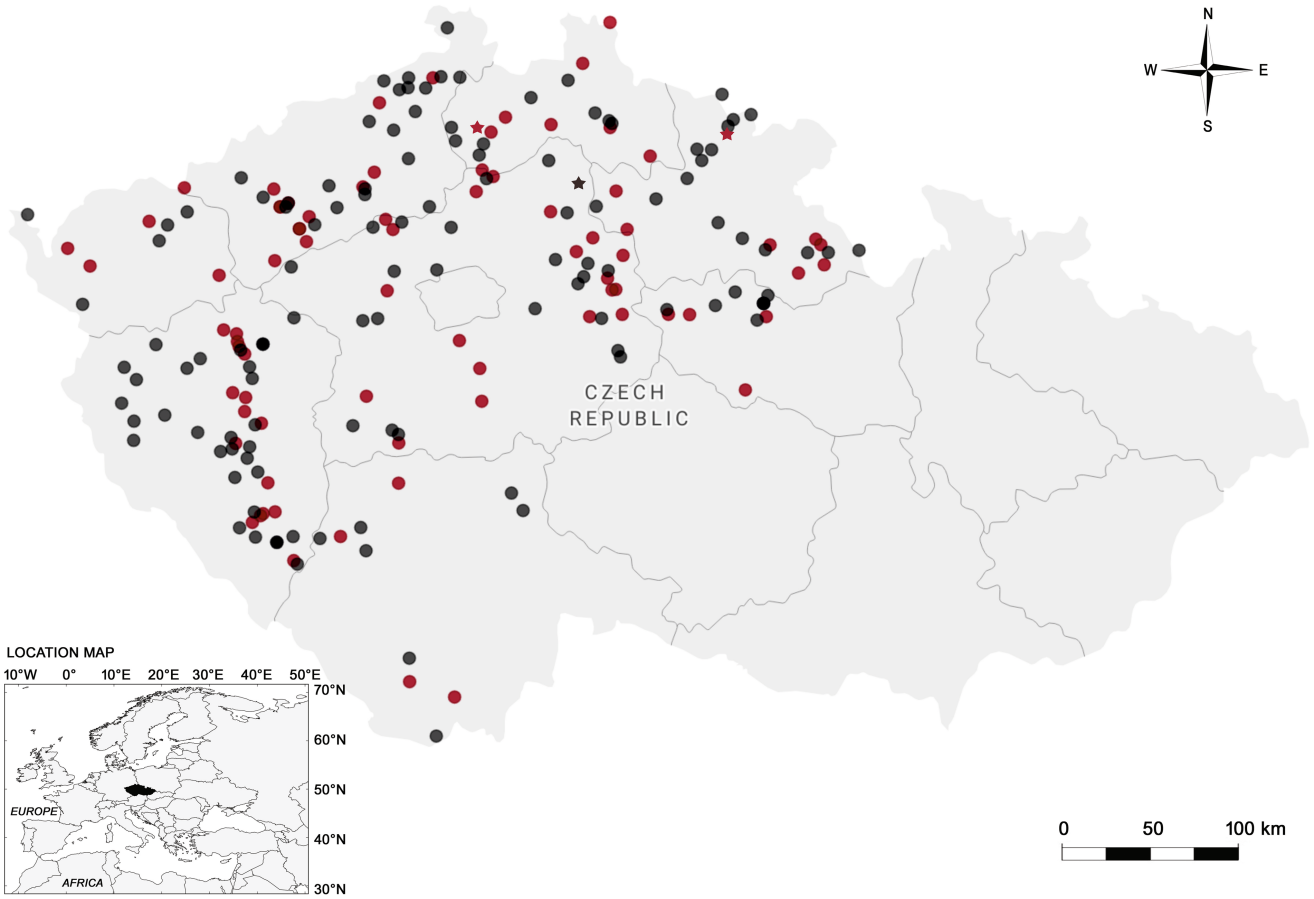
Map of the Czech Republic showing the sampling sites and regions (outlined areas) where red foxes (circles) and raccoon dogs (stars) were collected. Negative samples are represented in black color, while positive in red.

The whole intestinal tracts of 200 animals (197 red foxes and three raccoon dogs) were obtained during the monitoring on rabies and *Echinococcus* in 2019 from nine regions of the Czech Republic (Figure 1). These samples were sent to the State Veterinary Institute Prague and approximately 5 g of the contents of large intestine were taken and examined by flotation centrifugation coprological method according to Breza (30) using a Leica DMLB optical microscope with a Leica DFC420 digital camera (Leica Microsystems, Wetzlar, Germany) and concentrated sporocysts were transferred directly from glass slide by pipette to 2 mL Eppendorf safe-lock tubes with InhibitEX buffer. Only samples of 50 red foxes and one raccoon dog positive to *Sarcocystis* spp. were used for the molecular identification. Total genomic DNA (gDNA) was extracted from purified sporocysts using a QIAamp^®^ Fast DNA Stool Mini Kit (Qiagen, Hilden, Germany) according to the manufacturer’s instructions, except disruption of the sporocysts mixed with InhibitEX buffer with glass beads. The eluted DNA was kept at −20°C until further use.

The nested PCR (nPCR) method was used to amplify a fragment or partial sequence on the *cox1* gene from the collected DNA samples. Ten species-specific primer pairs for detection of *Sarcocystis* spp. using cattle (*Bos taurus*), goat (*Capra hircus*), horse (*Equus caballus*), pig, and sheep (*Ovis aries*) as intermediate hosts were utilized (see Table 1). Two of the tested *Sarcocystis* spp. (i.e., *S. hominis* and *S*. *suihominis*) employ humans as definitive hosts (3). The first round of amplification was carried out with a reaction mixture of 25 μL comprising 12.5 μL of DreamTaq PCR Master Mix (Thermo Fisher Scientific Baltics, Vilnius, Lithuania), 4 μL of DNA template, 0.5 μM of both forward and reverse primers, and nuclease-free water added up to 25 μL. nPCR was carried out using a Veriti 96-Well Thermal Cycler (Applied Biosystems, Foster City, CA, United States). The thermal cycling conditions began with 5 min at 95°C, followed by 35 cycles of 35 s at 94°C, 45 s at the species-specific annealing temperature (depending on the primer pair), and 60 s at 72°C, finishing with 5 min at 72°C. In the second round of amplification, 2 μL of the first round PCR product, 12.5 μL of DreamTaq PCR Master Mix, 0.5 μM of each internal primer specific to the species, and nuclease-free water were added up to 25 μL. Positive and negative controls, including nuclease-free water as a negative control and positive controls with previously acquired DNA samples from the sarcocysts of the corresponding *Sarcocystis* spp., were used for both rounds of nPCR. To visualize amplified products, 1% agarose gel (Thermo Fisher Scientific Baltics, Vilnius, Lithuania) electrophoresis was used. Obtained gel was documented using a BioDocAnalyze (Biometra, Gottingen, Germany) system.

**Table 1:** Data of the PCR primers of *Sarcocystis* species using farm animals as intermediate hosts used for the isolates from red fox and raccoon dogs in the Czech Republic.

Species	Primer				
	Name	Orientation	Sequence (5′-3′)	Ta, °C	bp
*S. arieticanis*	V2arie1^1^	Forward	CTCTTTGCCGTAGATTCGCTAGTTA	63	884
	V2arie2^1^	Reverse	CAAAGATCGGTAGATATCCAATGC		
	V2arie3^1^	Forward	TAGTTCTTGGCCTGGCTATTCTT	59	371
	V2arie4^1^	Reverse	CTGACCTCCAAAAACTGGCTTAC		
*S. bertrami*	V2ber3^1^	Forward	GTACTACCTCCTTCCAGTCGGTTC	57	605
	V2ber6^2^	Reverse	ACGACCGGGTATCCACTTCA		
	V2ber7^2^	Forward	CCCCACTCAGTACGAACTCC	59	381
	V2ber8^2^	Reverse	ACTGCGATATAACTCCAAAACCA		
*S*. *capracanis*	VocaF^1^	Forward	GTAAACTTCCTGGGTACTGTGCTGT	60	531
	VocaR^1^	Reverse	CCAGTAATCCGCTGTCAAGATAC		
	V2ca3^3^	Forward	ATACCGATCTTTACGGGAGCAGTA	63	330
	V2ca4^3^	Reverse	GGTCACCGCAGAGAAGTACGAT		
*S. cruzi*	V2cr1^1^	Forward	TACAATGTGCTGTTTACGCTCCA	61	777
	V2cr2^1^	Reverse	GCAATCATGATAGTTACGGCAGA		
	V2cr5^PS^	Forward	GGCCATTATATTCACGGCTTTA	57	251
	V2cr6^PS^	Reverse	GGCCGCCAAAAACTACTTTACT		
*S. heydorni*	V2hey1^PS^	Forward	TGGCCTCCTGGTTCTAGGC	57	354
	V2hey2^PS^	Reverse	CCATACCAAGGTGCCCAATATC		
	Shey3^PS^	Forward	AGTGTGCTCGGGTCGGTTA	55	329
	Shey4^PS^	Reverse	AACACCGCCTTACTGCCTACC		
*S. hircicanis*	V2hirici1^3^	Forward	CCGTAGATGCCATGGGTACTT	59	868
	V2hirici2^3^	Reverse	GTAGATATCCAGTGACGTGGTGAG		
	V2hirici3^4^	Forward	GCCTGGGTATTCTAGGACTGAGTAG	59	354
	V2hirici4^4^	Reverse	CGAAAACTGCTCTACCGCTCA		
*S. hominis*	GaHoEF^5^	Forward	TCTCTGGTTTTGGTAACTACTTCGT	65	551
	GaHoER^5^	Reverse	CAGACACTGGGATATAATACCGAAC		
	GaHoEF2^PS^	Forward	CATTGGCTGGACTCTCTATGCT	59	238
	GaHoER2^PS^	Reverse	AAATATCGGCAGGGTAATTATCAA		
*S. miescheriana*	V2mie3^1^	Forward	CTTGGTTCAACGTTACTCCTCCA	57	701
	V2mie2^1^	Reverse	GCCCAGAGATCCAAATCCAG		
	V2mie5^2^	Forward	TCCTCGGTATTAGCAGCGTACTG	55	338
	V2mie6^2^	Reverse	ATTGAAGGGCCACCAAACAC		
*S. suihominis*	V2su5^PS^	Forward	CAACGTGTACTTTACCATGCAC	55	590
	V2su6^PS^	Reverse	AGCCGGGCAGAATCAGAATA		
	V2su7^PS^	Forward	GTATGGCTAATCCACTCCGTAA	57	338
	V2su8^PS^	Reverse	GCATCATAAAAACCAAAGTTGAG		
*S. tenella*	V2te1^1^	Forward	GAGCGGTGAACTTCTTAGGAACC	61	537
	V2te2^1^	Reverse	CCCAATAATCCGCTGTTAACGTA		
	V2te3^PS^	Forward	CGATATGGAATTTAGTTTTGGATTG	61	288
	V2te4^1^	Reverse	ATAGTCACGGCAGAGAAGTAGGAC		

bp, base pairs; Ta, annealing temperature.

References: ^1^ Strazdaitė-Žielienė et al. (31), ^2^ Baranauskaitė et al. (32), ^3^ Marandykina-Prakienė et al. (33), ^4^ Prakas et al. (34), ^5^ Prakas et al. (35), ^PS^ Primers designed during the present study.

Obtained PCR samples were purified using phosphatase FastAP and exonuclease ExoI (Thermo Fisher Scientific Baltics, Vilnius, Lithuania). Positive PCR samples were subjected to sequencing performed using a Big-Dye^®^ Terminatorv3.1 Cycle Sequencing Kit (Thermo Fisher Scientific Baltics, Vilnius, Lithuania) and a 3500 Genetic Analyzer (Applied Biosystems, Foster City, CA, United States), following the manufacturer’s recommendations. After obtaining the *cox1* sequences of *Sarcocystis* spp., nucleotide BLAST function was employed to compare them with similar ones available in NCBI GenBank.^1^ The phylogenetic analysis was carried out by the help of MEGA v11.0.13 (36). Multiple alignments were obtained using ClustalW algorithm. The Kimura 2-parameter evolution model with a gamma distribution (K2 + G) was chosen as the best fit to the data for all analyses. Phylogenetic trees were rooted on *S. hirsuta*. The robustness of phylogenetic trees was tested using bootstrap test with 1,000 replicates. The map was drawn using Datawrapper server.^2^

## Results

In total, 75 out of 197 (38.1%; 95% CI = 31.45-45.15) intestinal mucosa samples of the red fox and two out of three (66.7%; 95% CI = 13.54-98.30) of the raccoon dog were found to be *Sarcocystis*-positive by light microscopy. Of these, 50 samples of red fox and one of raccoon dog were used for further molecular characterization, which resulted in the identification of five species in the red fox: *S*. *arieticanis*, *S*. *capracanis*, *S. cruzi*, *S*. *miescheriana*, and *S. tenella*. At least one *Sarcocystis* species isolate was present in 21 out of 51 (41.2%) red foxes, while the PCR was negative for the sole raccoon dog.

*Sarcocystis arieticanis*, *S*. *capracanis*, *S. cruzi*, and *S*. *miescheriana* were amplified by using nPCR, and amplified fragments were visible only after the second round of nPCR, whereas *S. tenella* was observed using the direct PCR. Sequences of these five species obtained in our study (GenBank accession numbers: PP358805–PP358830) were compared to those of the same and closely related *Sarcocystis* spp. available in GenBank (see Table 2). When comparing the sequences found in this study, the highest intraspecific variation was found for *S*. *miescheriana* (94.3–99.7%). Notably, the obtained intraspecific and interspecific genetic variability values for all detected species did not overlap, thus showing that species have been correctly identified.

**Table 2:** Molecular information of the *cox1* sequences of five *Sarcocystis* species found in the large intestine of red fox (*Vulpes vulpes*) from the Czech Republic.

	*S*. *arieticanis*	*S*. *capracanis*	*S*. *cruzi*	*S*. *miescheriana*	*S*. *tenella*
Sequence length (base pairs)	325	284	207	315	491
GenBank accession numbers	PP358829	PP358830	PP358817–PP358822	PP358823–PP358828	PP358805–PP358816
Intraspecific similarity of sequences in the present study	*	*	99.0–100%	94.3–99.7%	98.6–100%
Similarity with other isolates of the same species	97.2–99.7%**	96.8–99.3%	95.2–100%	93.0–99.7%	95.3–100%
Interspecific similarity with the most related species	*S*. *hircicanis* 87.2–87.7%	*S. tenella* 91.8–93.2%	*S*. *levinei* 90.8–91.8%	*S*. *rangiferi* 75.6–78.9%	*S*. *capracanis* 89.2–91.5%

*Intraspecific similarity values cannot be determined due to only one obtained sequence for this species, ** excluding *S. arieticanis* isolated from domestic sheep in Egypt (MH413047-8) (92.6–93.5%).

*Sarcocystis* spp. coinfections occurred in the large intestine of the red fox. Four different parasite species (*S*. *arieticanis*, *S*. *capracanis*, *S. cruzi*, and *S. tenella*) were found in a single specimen of red fox, *S. cruzi*/*S. tenella* and *S. cruzi*/*S*. *miescheriana* were identified in two separate foxes, while the remaining 18 samples were confirmed with a single *Sarcocystis* species. Among the molecularly confirmed species, *S. tenella* was the most prevalent, whereas the detection rates of other four *Sarcocystis* spp. were lower, and two parasite species were only detected in a single fox (Table 3).

**Table 3:** Infection parameter (prevalence) of the five *Sarcocystis* species molecularly confirmed in the red fox (*Vulpes vulpes*) from the Czech Republic.

Species	Intermediate host	Number of positive samples	Prevalence (%)	95% confidence intervals of prevalence
*S*. *tenella*	Sheep	12	23.5	13.4–37.2
*S*. *cruzi*	Cattle	6	11.8	5.3–23.4
*S*. *miescheriana*	Pig/wild boar	6	11.8	5.3–23.4
*S*. *arieticanis*	Sheep	1	2.0	0.1–10.4
*S*. *capracanis*	Goat	1	2.0	0.1–10.4

**Figure 2:**
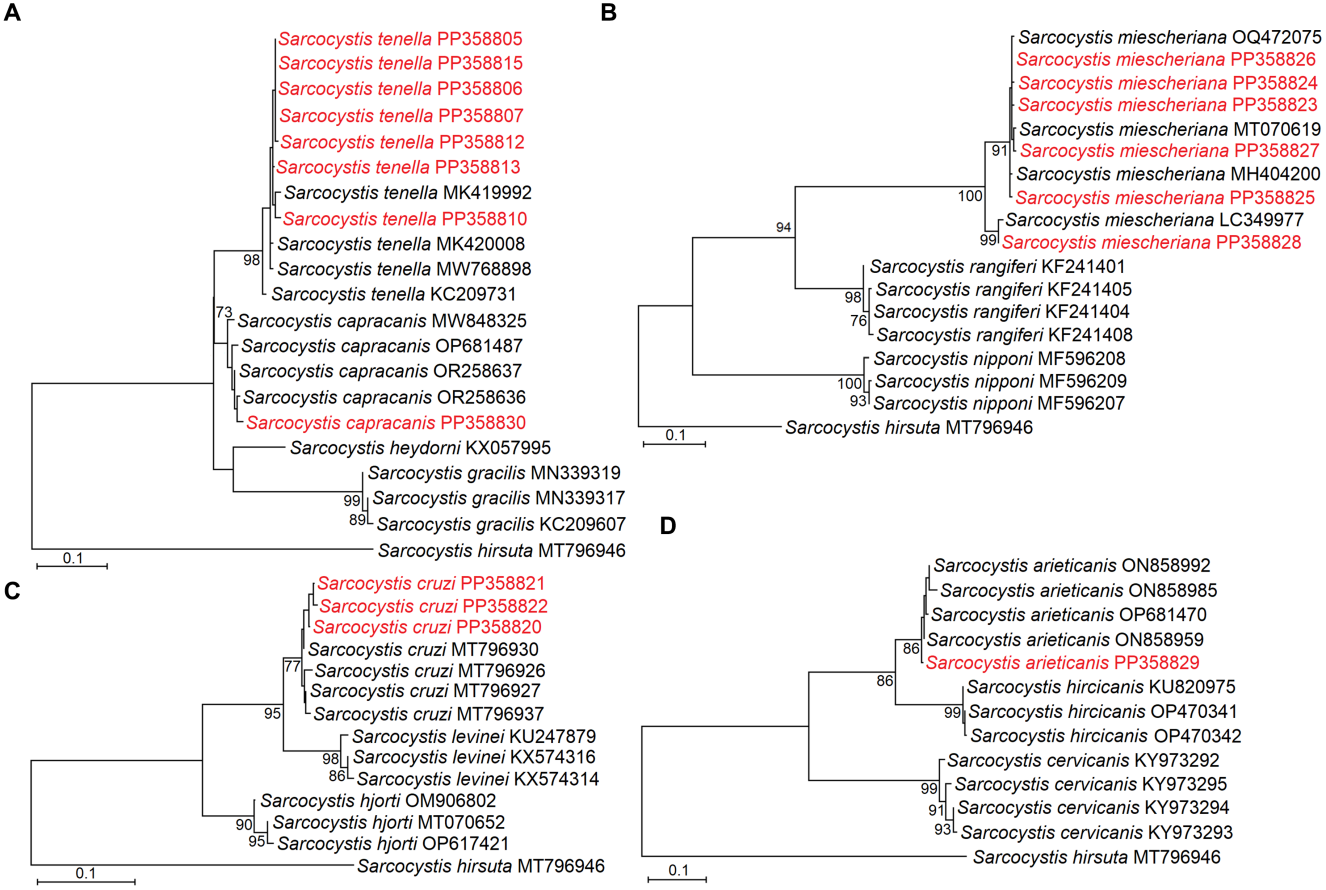
Phylogenetic trees based on *cox1* sequences showing placement of *Sarcocystis tenella* and *S*. *capracanis* **(A)**, *S*. *miescheriana* **(B)**, *S. cruzi* **(C)**, and *S*. *arieticanis* **(D)**. Sequences representing different haplotypes identified in the present study are marked in red.

The phylogenetic analysis also confirmed the identification of five *Sarcocystis* species in the large intestines of red foxes. Based on phylogenetic results close relationship was established between *S*. *capracanis* and *S. tenella* (Figure 2A), *S*. *arieticanis* was a sister species to *S*. *hircicanis* (Figure 2D). Additionally, *S. cruzi* was sister taxa to *S*. *levinei* (Figure 2C), while a relatively high genetic distance was determined comparing *S*. *miescheriana* with other *Sarcocystis* spp. (Figure 2B).

The sequences generated in the present study were submitted to the GenBank database under the accession numbers: PP358805–PP358816; PP358817–PP358822; PP358823–PP358828; PP358829; PP358830.

## Discussion

The red fox and raccoon dog are one of the most widespread and invasive wild terrestrial carnivores that have been involved in the life cycle of *Sarcocystis* as either intermediate or definitive hosts (37–40). In the present case, the red fox might serve as definitive host of five *Sarcocystis* spp., which use farmed animals (e.g., sheep, cattle, pig/wild boar, and goat) as intermediate hosts (41, 42). On the other hand, the raccoon dog was parasitized by sporocysts of a *Sarcocystis* species, although their molecular characterization resulted in negative PCR and require the use of other sets of primers. Therefore, this is the first report of the red fox as definitive host of *Sarcocystis* spp. molecularly characterized in the Czech Republic.

The examination of the small intestinal mucosa is a common technique for detecting apicomplexans in individual animals (1, 10, 43–45), since it minimizes the risk of reporting sporocysts coming from the prey (“passage sporocysts”) and allows the finding of higher number of oocysts/sporocysts, which are released in small amounts in feces (18). In this survey, the presence of developmental stages of *Sarcocystis* spp. in the anterior large intestine demonstrated that this part of the digestive tract is also useful for obtaining epizootiological data on these parasites.

In this study, the values of prevalence in the red fox were higher after the examination of the large intestinal mucosa in comparison to those of molecular analysis. During the first method, the whole *Sarcocystis* richness is pooled together and might generate overestimated prevalence, while in the second method each species is individually identified, thus resulting in more particular values. The microscopical and molecular approaches are mandatory for the study of these protozoans, although the latter is the best to categorize the *Sarcocystis* spp. (46). Moré et al. (18) found similar prevalence in the red fox (38.0%) and lower in the raccoon dog (52.6%), whereas Prakas et al. (26) reported lower prevalence (20.0%) of apparently various *Sarcocystis* spp. and especially of *S. rileyi* in the red fox and raccoon dog from Lithuania. The contrasting results between surveys should be taken cautiously since data come from different number of samples, climatic seasons, age of hosts, locality, availability of intermediate hosts, parasitological skills of the observer, and proper molecular analysis. Unfortunately, the sporocysts in the raccoon dog were not molecularly characterized and their identity remains uncertain. The present values of prevalence are determined for the first time for five *Sarcocystis* spp. in the red fox.

Out of the five species herein molecularly identified, *S*. *arieticanis* predominantly occurs in sheep [e.g., (33, 47)], *S*. *capracanis* in goat [e.g., (33, 48)], *S. cruzi* in cattle [e.g., (49, 50)], *S*. *miescheriana* in wild boar [e.g., (51–53)], and *S. tenella* also in sheep [e.g., (47, 54)]. The role of canids (e.g., dog, jackal, raccoon dog, red fox, and gray wolf) as definitive hosts of these *Sarcocystis* spp. has been previously confirmed (18). Particularly, the red fox is known as the main scavenger of wildlife (55) and commonly feeds on pigs or wild boars, so its role as definitive host for these five *Sarcocystis* spp. is possible. The occurrence of these parasite species is likely linked to the presence of canid hosts and the close trophic interaction between predator and prey, as already stated (52).

Previously, DNA of zoonotic *S. hominis* was detected in a single small intestine mucosal sample of European pine marten (*Martes martes*) from Lithuania (56). Fortunately, none of the five *Sarcocystis* spp. found in the present investigation is known to be zoonotic. However, the diagnostic and monitoring of *Sarcocystis* and other parasites in farm animals should be imperative, since, for example, wild boar might be infected by *S*. *suihominis* (52) and cattle by *S. hominis* and *S*. *heydorni* (16, 36, 57), which actually are zoonotic and might be potentially transmitted to humans through the consumption of raw or undercooked meat. On the other hand, domestic pigs experimentally infected with *S*. *miescheriana* showed symptoms as reduced weight gain, cutaneous purpura, dyspnea, muscle tremors, abortion, and death (52). The transmission of *Sarcocystis* spp. through canids to farm animals is more dangerous and cause similar symptoms than those mentioned, as well as fever, anemia, and reduction in milk yield (1).

Since the present survey was based on the use of species-specific primers, some *Sarcocystis* spp. were absent from the analysis, like *S*. *capreolicanis*, *S. gracilis*, and *S. rileyi*, that use wild animals as intermediate hosts (1, 26, 40). The *cox1* gene clearly differentiated the closely related *Sarcocystis* spp. in the present study and being very useful for those species having ungulates as intermediate hosts (16, 50). If the complete role of canids in the life cycle of *Sarcocystis* pretends to be elucidated, samples from more farm animals and wildlife should be examined and characterized.

The presence of *Sarcocystis* in the intermediate hosts might lead to economic losses or represent a zoonotic risk for humans (8). However, most of studies lacks proper molecular characterization of the species and thus their proper diagnosis and assessment of their importance is overlooked. Therefore, the present findings suggest a potential role of red fox populations in the transmission of *Sarcocystis* to wild and farmed animals in the study area.

The occurrence of sporocysts in the red fox and raccoon dogs indicates that both wild animal species might be spreading and transmitting these developmental stages (via feces, water, or food) to farm or zoo animals, but probably also to breeders or the staff from zoological gardens. The human activities and destruction of habitats produce a more frequent interaction between canids with farm animals that might produce higher prevalence, as occurred with the European gray wolf and its prey in Central Europe (58), although mesopredators may maintain *Sarcocystis* life cycles in the absence of the suitable definitive host (18). Particular attention should be paid to the handling process of hunters and/or shepherds in leaving carcasses or viscera infected with *Sarcocystis* on the ground and that might promote the dissemination of the parasite (59), because after feeding on infected meat, canids begin shedding sporocysts in the environment that might be infective for farmed and wild animals. If possible, reduce the free access of canids to pasture, feeders and water sources in the farms and the exposure of farm animals to feces of wild canids. Frequently, the species of *Sarcocystis* are non-pathogenic for farm animals, but when sarcocysts are large and evident, significant losses occur in the animal husbandry industry (60).

This is the first study where the potential role of the red fox and raccoon dogs as spreaders of *Sarcocystis* to farm animals in the Czech Republic is shown. However, more data from other definitive hosts and countries are needed to fulfil the missing data on the main definitive hosts or environmental samples around farms or zoological gardens. Moreover, the huge populations of red fox and raccoon dogs need to be controlled by hunting to avoid the transmission of these and other parasites.

## Conclusion

The proper morphological molecular characterization of *Sarcocystis* spp. is extremely important to identify and thus take the actions to control their spreading through the environment and hosts and ensure food safety and avoid economic losses. This could lead to proper prevention for breeders and avoid potential risks for their animals, as well as to detect pathogenic or zoonotic species that might be transferred to humans. The use of species-specific primers provides a fast and easy method for screening multiple samples for a particular *Sarcocystis* species. However, it is necessary to use more general primers or cloning of PCR products and sequence a few samples in order to detect a mixed infection with unexpected species, not targeted by species-specific primers.

## Data availability statement

The datasets presented in this study can be found in online repositories. The names of the repository/repositories and accession number(s) can be found in the article/supplementary material.

## Ethics statement

Ethical approval was not required for the study involving animals in accordance with the local legislation and institutional requirements because animals were killed by the hunters.

## Author contributions

OM: Conceptualization, Formal analysis, Funding acquisition, Investigation, Methodology, Writing – original draft, Writing – review & editing. NG: Formal analysis, Methodology, Writing – review & editing. DB: Formal analysis, Methodology, Writing – review & editing. DG-S: Conceptualization, Formal analysis, Supervision, Writing – original draft, Writing – review & editing. PP: Conceptualization, Formal analysis, Methodology, Writing – original draft, Writing – review & editing.

## Footnotes

^1^http://blast.ncbi.nlm.nih.gov/, accessed on 14 February 2023.

^2^ https://www.datawrapper.de/
